# Giant cavernous hemangioma of the eleventh rib

**DOI:** 10.1186/s13019-019-0919-6

**Published:** 2019-05-22

**Authors:** Heping Huang, Chengdong Ning, Yu Pan

**Affiliations:** Department of Cardiothoracic Surgery, The Lu’an affiliated Hospital, Anhui Medical University, No. 21, west wanxi road, Jin’an district, The Lu’an city, 237005 Anhui Province China

**Keywords:** Cavernous hemangioma, Thoracotomy, Tumor, Diagnosis

## Abstract

**Background:**

Cavernous hemangioma of the rib is extremely rare benign vascular tumor. It is difficult to diagnose in time because both invasive and noninvasive examinations usually fail to distinguish it from other tumors of the rib and other bones.

**Case presentation:**

We described an asymptomatic 44-year-old woman with cavernous hemangioma of the rib that was incidentally discovered in the bathing. The tumor was completely resected by minithoracotomy through posterolateral incision. The pathological tissue was diagnosed as a cavernous hemangioma composed of thin-walled blood vessels and red blood cells.

**Conclusions:**

We reported this case of giant cavernous hemangioma of the rib for its extremely rare occurrence. The preoperative diagnosis is a challenge both clinically and radiologically, and difficult to distinguish this tumor from other tumors of the rib or long bones.

## Background

Hemangioma is not a neoplasm, but rather a congenital venous malformation with the potential to develop in all part s of the body. They are predominantly found in the spine and skull and are uncommonly observed in the ribs or long bones [1, 2]. Cavernous hemangioma of the rib is extremely rare benign vascular tumor, which should be considered in the differential diagnosis of rib tumors, especially in asymptomatic patients.

However, we describe an extremely rare case of a cavernous hemangioma of the rib which was found accidentally in a female patient, the preoperative investigations, and the surgical treatment.

## Case presentation

**Fig. 1:**
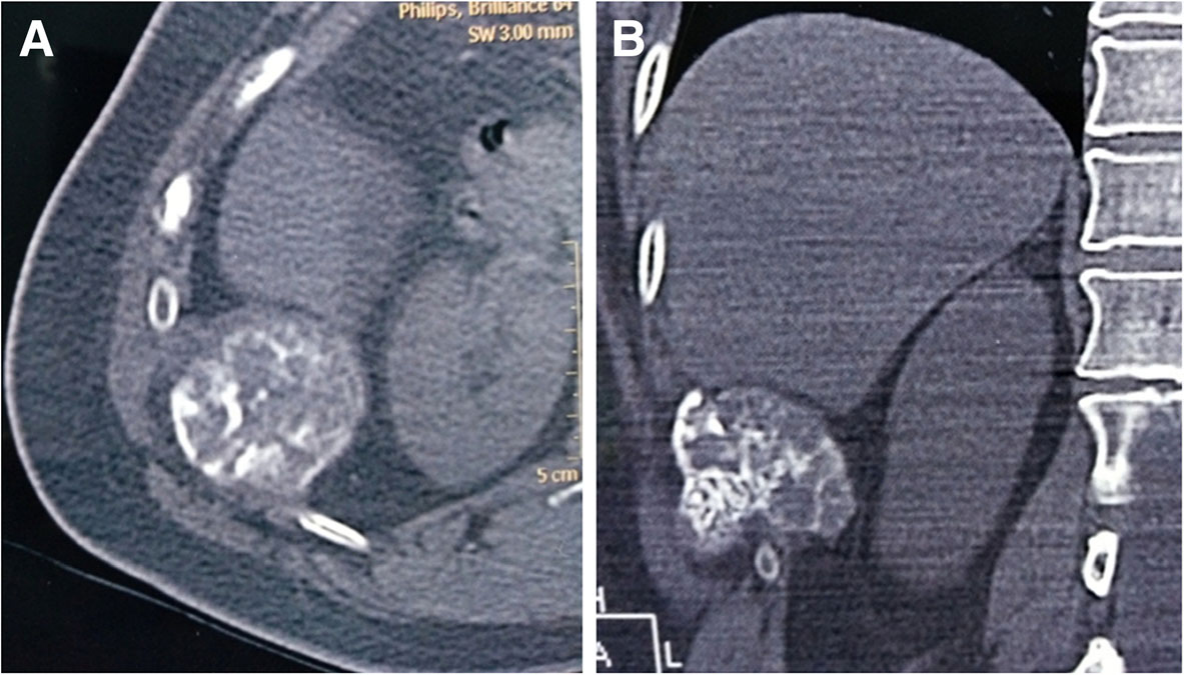
Chest computed tomography (CT), showing a tumor in the right eleventh rib(**a**), near the right lower lobe of liver (**b**), and an osteolytic eccentric expansive mass with sunburst calcification and focal cortical disruption

An asymptomatic 44-year-old female with no medical history or history of trauma to the chest wall was admitted due to a right chest wall mass which was incidentally discovered in the shower. Chest computed tomography (CT) demonstrated a tumor, measuring 8.5 cm in diameter. Osteosclerosis was present on the top of the lesion along with calcification in different places and thickening on the nearby parietal pleura and diaphragm (Fig. 1a,b).

**Fig. 2:**
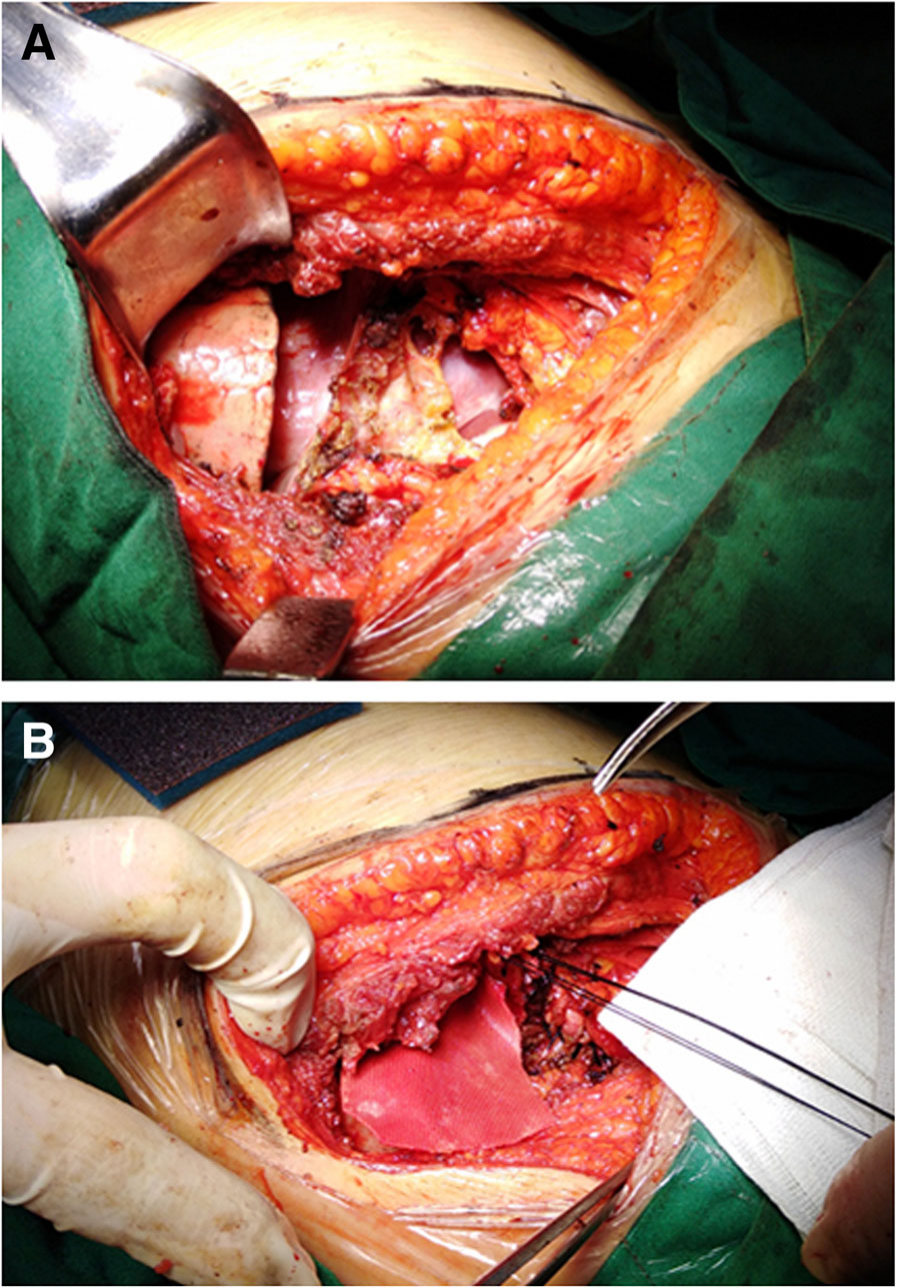
Intraoperative photographs showed the large chest wall defect (**a**) caused by rib resection and performance of a polyester patch draft (**b**)

The laboratory investigation including serum tumor marker levels and routine hematologic, blood biochemistry results were normal. The patient underwent right lateral minithoracotomy in which a partial excision of the rib was performed, the intraoperative exploration showed the diaphragm was closely adhered to the giant mass. The large chest wall defect caused by rib resection was reconstructed by performing a polyester patch draft (Fig. 2a,b). When thoracic incision was closed, the right lung was insufflated by anesthetist to expel gas in the right thoracic cavity. No thoracic close drainage was placed to reduce postoperative pain and complications.

**Fig. 3:**
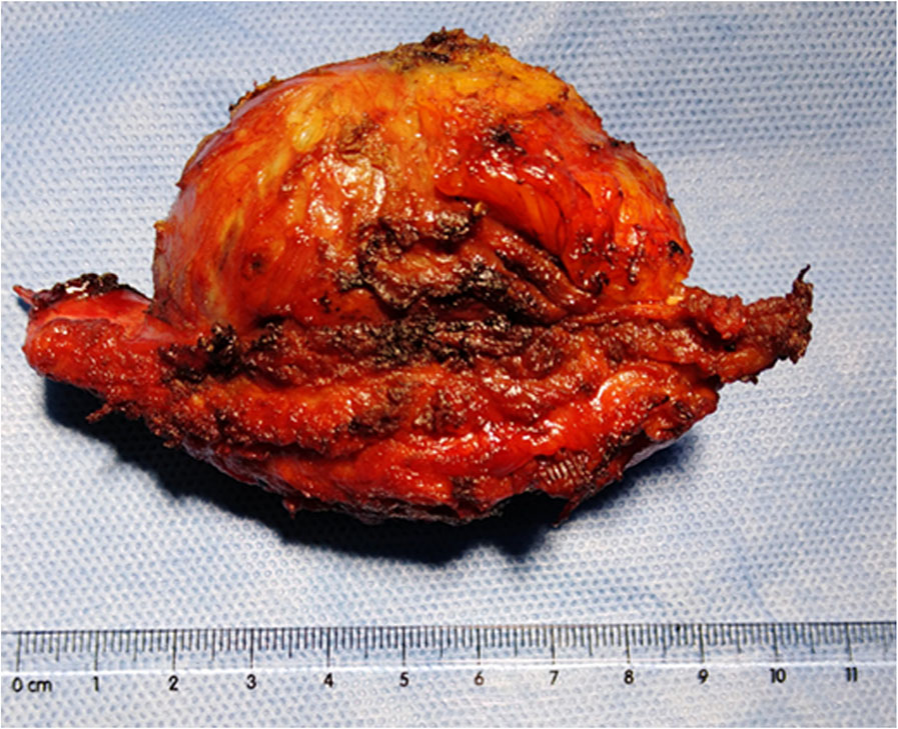
Macroscopic appearance of resected material

**Fig. 4:**
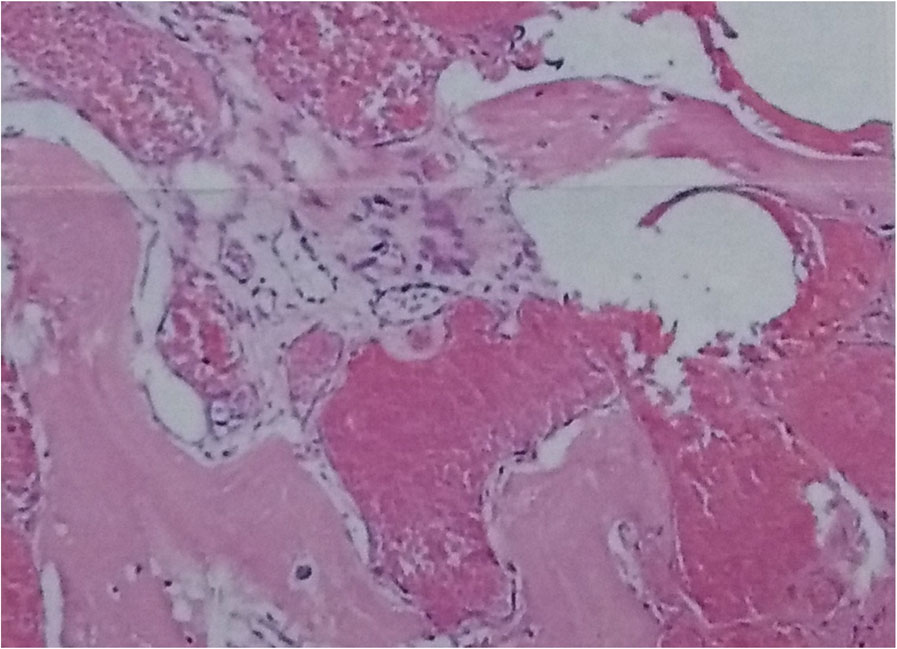
Histopathologic examination of resected material showing the mass consists of thin-walled blood vessels with single layer of endothelial-cell lining containing red blood cells. (H & E 100×)

The patient had an uneventful recovery and discharged on the sixth postoperative day. Five months after the operation, she was doing well, without any evidence of local recurrence. A definite diagnosis of cavernous hemangioma was made based on histopathology examination results of the resected mass (Fig. 3). The mass was composed of thin-walled blood vessels with dilated channels containing red blood cells and lined by a single layer of endothelial cells (Fig. 4).

## Discussion

Most cases of bone hemangiomas develop in the vertebral body or the skull. Hemangioma of the rib is rare, both as a rib tumor and as a bone Hemangioma [3]. It is mostly detected incidentally as it is generally asymptomatic [4], so did our case. However, about 50% of rib tumors are malignant, and it is difficult to distinguish a rib hemangioma from a malignant tumor such as a chondrosarcoma, metastatic tumor, or multiple myeloma [5].

Hemangioma is a benign neoplasm of blood vessels that can occur throughout the body. Histologically, there are four types of hemangiomas: cavernous, capillary, venous, and mixed type [6]. Cavernous hemangiomas are the most common and account for up to 50% of all hemangiomas. They consist of dilated vessels lined by a single layer of endothelial cells surrounded by a fibrous stromal layer. Most cavernous hemangiomas involve the medullary and intracortical portion of the bone.

Bone hemangiomas can present with different features on scans obtained by using varying imaging techniques. 18 F-FDG PET can detect the elevated glucose metabolism of cells, which is widely used for differentiation between benign and malignant neoplasms. Malignant lesions tend to be 18 F-FDG avid, however, benign lesions generally show lower 18F-FDG avidity [7]. Choi et al. reported that the mean SUVmax values in the benign rib lesions were 2.5 ± 1.1 [8]. Preoperative 18 F-FDG PET examination in our case was not performed because of the patient’s poor family economy condition.

Preoperative diagnosis is not always possible due to overlapping radiological features between benign and malignant lesions. It is difficult to make a preoperative definite diagnosis of the chest wall tumors by imagery alone. Therefore, most of the patients with a rib hemangioma undergo rib resection. However, if CT scans show an osteolytic expansive lesion containing sunburst calcifications with low 18F-FDG avidity, a diagnosis of rib hemangioma should be considered [9].

It is the treatment of choice to make a surgical excision of the rib while the histological examination reveals the diagnosis. Excision of rib hemangiomas is a safe procedure with no reported complication after removal or recurrence. Generally, needle biopsy should be avoided because of the risk of life-threatening bleeding or seeding the needle tract unless multiple myeloma or metastatic disease is highly suspected [3].

In addition, temporary embolization using gel foam in rib hemangioma causes shrinkage in size and vascularity of the lesion drastically further easing the excision of the lesion.

We have reviewed 36 cases of rib hemangiomas available in the literature from August 1994 to November 2018 (Table 1). Among these limited cases, the woman was far more than the man in sexual distinction. Meanwhile, rib hemangioma is more common in middle-aged patients, in which mean and standard deviation values of the age is 47.0 ± 16.9.Futhermore, the sickness incidence of the seventh and eighth ribs was significantly higher than the others in lesion region. Nevertheless, there is not much difference between the left and the right side of body in rib lesion position.

**Table 1:** Characteristics of patients

Sex		Age(years)	Location		Rib position		
Female	Male	Mean and standard deviation	Left	Right	Seventh	Eighth	Others
21	15	47.0 ± 16.9	19	17	12	7	17

Others: including first,third,forth,fifth,sixth,ninth and tenth rib

## Conclusions

We reported this case of cavernous hemangioma for its extremely rare occurrence in the eleventh rib, which is the false and floating rib and relatively small and delicate. Preoperative diagnosis remains a challenge both clinically and radiologically. It is still difficult to distinguish the disease from other tumors in the rib. Furthermore, surgical resection provides materials for histopathologic diagnosis.

In the future, we hope more cases of rib hemangiomas will be investigated and explored to find out reasons for gender and location differences of rib hemangioma.

## Abbreviations

CT: computed tomography
H & E: hematoxylin and eosin
